# Buffer Components Incorporate into the Framework of Polyserotonin Nanoparticles and Films during Synthesis

**DOI:** 10.3390/nano12122027

**Published:** 2022-06-13

**Authors:** Keuna Jeon, Justin Andrei Asuncion, Alexander Lucien Corbett, Tiange Yuan, Meera Patel, Nesha May Octavio Andoy, Christian Titus Kreis, Oleksandr Voznyy, Ruby May Arana Sullan

**Affiliations:** 1 Department of Physical and Environmental Sciences, University of Toronto Scarborough, 1065 Military Trail, Toronto, ON M1C 1A4, Canada; k.jeon@utoronto.ca (K.J.); justin.asuncion@mail.utoronto.ca (J.A.A.); alexander.corbett@mail.utoronto.ca (A.L.C.); gary.yuan@mail.utoronto.ca (T.Y.); meera.patel@mail.utoronto.ca (M.P.); nesha.andoy@utoronto.ca (N.M.O.A.); christian.kreis@utoronto.ca (C.T.K.); o.voznyy@utoronto.ca (O.V.); 2 Department of Chemistry, University of Toronto, 80 St. George St., Toronto, ON M5S 3H6, Canada

**Keywords:** polyserotonin nanoparticles, polyserotonin films, nanomechanical properties of polyserotonin, elasticity of polyserotonin, bioinspired nanomaterials, atomic force microscopy (AFM), AFM quantitative imaging (QI)

## Abstract

Polyserotonin nanoparticles (PSeNP) and films are bioinspired nanomaterials that have potential in biomedical applications and surface coatings. As studies on polyserotonin (PSe) nanoparticles and films are still in their infancy, synthetic pathways and material development for this new class of nanomaterial await investigation. Here, we sought to determine how different buffers used during the polymerization of serotonin to form nanoparticles and films impact the physicochemical properties of PSe materials. We show that buffer components are incorporated into the polymer matrix, which is also supported by density functional theory calculations. While we observed no significant differences between the elasticity of nanoparticles synthesized in the different buffers, the nanoscale surface properties of PSe films revealed dissimilarities in surface functional groups influenced by solvent molecules. Overall, the results obtained in this work can be used towards the rational design of PSe nanomaterials with tailored properties and for specific applications.

## 1. Introduction

Various classes of materials—polymeric, inorganic, and lipid-based—have been used as functional nanomaterials for a broad range of applications. Nanomaterials are known for their versatility, i.e., synthesis conditions can be defined to obtain a desired size, charge, and shape [1]. Many of these nanomaterials also allow for the fine tuning of their surface functional groups to address the limitations of current therapeutics [2,3]. For example, surface-modified quantum dots [4] were demonstrated to penetrate into biofilms at sites of infections, while polymersomes [5] were used to facilitate drug delivery into the cell nucleus.

Largely contributing to the versatility of organic nanomaterials is the presence of various functional groups that facilitate interaction with other molecules via multiple and different intermolecular forces, such as hydrogen bonding, π–π stacking, polar interactions, and even metal chelation [6,7]. In addition, external factors, such as pH, temperature, presence of ions, and nature of organic solvents also affect nanomaterial synthesis. For example, the effect of a solvent in the self-assembly of polydopamine was investigated and, depending on pH [8], solubility parameter [9], and buffer type [10], differences in self-assembly pathways were observed. When designing a nanomaterial, surface adhesive properties and material elasticity are also important considerations as these could influence cellular uptake, tissue penetration, and distribution, as well as other structural and functional properties [11,12].

In 2018, polyserotonin nanoparticles (PSeNP) joined the class of bioinspired materials as a multifunctional nanomaterial with potential for biomedical applications [13]. Nakatsuka et al. first demonstrated PSeNP’s photothermal capability and its ability to serve as an effective anti-cancer drug nanocarrier [13]. Polyserotonin (Pse) was also found to reduce protein adhesive properties compared to polydopamine [13,14]. In 2021, our group developed a facile approach to controlling PSe particle size by tuning the initial reaction pH and the base used during synthesis [15]. A narrow size distribution was observed in the presence of amines in the synthesis buffer (Tris) compared to when there were no amine groups present (PBS). We further observed smaller PSeNP in a primary amine (Tris) than in a tertiary amine (Bicine) solvent. Recently, Zhou and coworkers demonstrated the potential utility of polyserotonin as a versatile coating (with a pH-responsive degradation profile) for anti-tumor therapy [16]. Considering the promise of PSeNP towards biomedical applications and/or as a universal surface coating, it is important to establish how different solvent systems affect their size, shape, and nanomechanical properties to further exploit and expand their potential for various applications.

## 2. Materials and Methods

### 2.1. Synthesis of Polyserotonin (PSe) Nanoparticles and Films

**Scheme 1:**
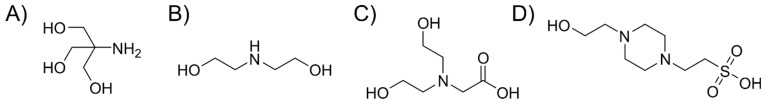
Chemical structures of the synthesis buffers: (**A**) trisaminomethane (Tris), (**B**) diethanolamine (DEA), (**C**) bis (2-hydroxylethyl)amino acetic acid (Bicine), and (**D**) 4-(2-hydroxylethyl)-1-piperazineethanesulfonic acid (HEPES).

Polyserotonin nanoparticles (PSeNP) were synthesized using four different buffers: (1) trisaminomethane (Tris), (2) 4-(2-hydroxylethyl)-1-piperazineethanesulfonic acid (HEPES), (3) diethanolamine (DEA), and (4) bis (2-hydroxylethyl)amino acetic acid (Bicine), all purchased from Sigma-Aldrich (Oakville, ON, Canada) (see Scheme 1 for chemical structures). Briefly, 100 mL of 10 mM buffer at pH 9 was placed in a 500 mL single-neck round-bottom flask and heated in a water bath to 60 °C. Serotonin hydrochloride (2 mg/mL) (Tokyo Chemical Industry America, Tokyo, Japan) was then added, stirred, and capped for 45 min. Subsequently, the mixture was purified by centrifugation (Eppendorf centrifuge 5804R) at 16,639 rcf at 4 °C for 5 min. Pellets were removed, while allowing the supernatant to further grow in the dark for an additional 24 h. The nanoparticles were then centrifuged out at 16,639 rcf at 4 °C for 1 h followed by triple washings with milli-Q water at 20,817 rcf at 4 °C for 15 min. In addition to nanoparticles, PSe films were also prepared by incubating coverslips in the supernatant for 24 h.

### 2.2. Characterization of PSe Nanoparticles and Films

Optical absorption spectra to quantify the growth of PSeNP were acquired using Ultraviolet-visible (UV-vis) spectrometer (Cary 60 UV-Vis, Agilent Technologies, Santa Clara, CA, USA). Polyserotonin nanoparticles were imaged with transmission electron microscopy (TEM, Hitachi H7500 with iTEM version 5.2 software, Tokyo, Japan) and analyzed with Image J to determine nanoparticle size. Global size distribution was also estimated using dynamic light scattering (DLS, NanoBrook Omni, Brookhaven Instruments, Holtsville, NY, USA) in Tris buffer (10 mM, pH 7.5). Surface zeta potential were determined using phase analysis light scattering (PALS, NanoBrook Omni, Brookhaven Instruments, Holtsville, NY, USA) using the same conditions as the DLS measurements. Free radical centers on PSeNP were analyzed via electron paramagnetic resonance (EPR) spectrum using a Bruker X-band CW EMX EPR spectrometer equipped with a 10” electromagnet and an ER 4123D resonator.

Contact angles (Droplet Smart Tech, Markham, ON, Canada) and surface energy of PSe films synthesized in different buffers were also determined. Briefly, 20 µL of pure diiodomethane (Alfa Aesar, Haverhill, MA, USA), glycerol (BioShop, Burlington, ON, Canada), and milli-Q water were manually dropped onto the films and imaged with a camera (0.3 Mp) to estimate the angles of each drop. Contact angles were analyzed using a program that was written in-house, and the surface energy was calculated using the Owens–Wendt–Rabel–Kaelbel (OWRK) model.

Nanomechanical characterization of PSeNP was performed using the quantitative imaging modality of the atomic force microscope (AFM, Nanowizard 4, JPK Instruments, Berlin, Germany). Samples for AFM imaging were prepared by drop casting the nanoparticles on glass coverslips coated with polyethyleneimine (PEI, Sigma-Aldrich, Oakville, ON, Canada). PointProbe Plus Non-Contact High resonance frequency cantilevers with gold coatings on the detector side (PPP-NCHAuD, Nanosensors, Neuchâtel, Switzerland) were used to measure the apparent Young’s modulus of elasticity of the nanoparticles in 10 mM Tris pH 7.5. Before elasticity measurement, the cantilevers were first UV cleaned for 10 min, and thermal tuning was performed in air to calibrate the cantilevers. This resulted in spring constants in the range of 39.1–47.4 N/m. An in-house Python script was used to analyze the sample topography and extract the Young’s modulus of PSeNP [17]. In addition, scratch tests were performed on PSe films using the same cantilevers, and the film thickness was determined using the JPK Image Processing software. For adhesion measurements (on PSe films), silicon nitride tips (MSCT-D, Bruker, Camarillo, CA, USA) were first functionalized with (3-aminopropyl)triethoxysilane (APTES), following the protocol described in Zimmermann et al. [18]. Thermal tuning of the functionalized cantilevers yielded spring constants in the range of 0.035–0.07 N/m. Force–distance curves were then acquired (sampling rate of 1 μm/s) in 10 mM Tris buffer at pH 9 and pH 7.5, and the adhesion force and work of adhesion were subsequently determined.

### 2.3. Density Functional Theory (DFT) Calculations of PSeNP

The density functional theory (DFT) calculations were conducted through CP2K with Perdew-Burke-Ernzerhof as the exchange correlation and conjugate gradients as the minimizer. Molopt DZP was selected as a basis set, along with Godecker-Teter-Hutter as the pseudopotentials. Each calculation was performed in a 20 × 20 × 20 Å^3^ unit cell. The grid cutoff value was set to 600Ry. Each molecule was fully relaxed individually through geometry optimization. The relaxed molecules were used for interaction calculations. The binding energy was calculated as follows:

*BE_AB_* = *E_AB_* − *E_A_* − *E_B_* (1)

where *BE_AB_* is the binding energy of *A* interacting with *B*, *E_AB_* is the energy of *A* interacts with *B*, *E_A_* is the energy for *A* after geometry optimization, *E_B_* is the energy for *B* after geometry optimization.

When performing the geometry optimization calculations, the structures of the monomers were adjusted based on the working pH and included an overall charge of the system for each pH calculated.

## 3. Results and Discussion

### 3.1. Buffer Components Impact PSeNP Size but Not Their Nanomechanical Properties

**Figure 1:**
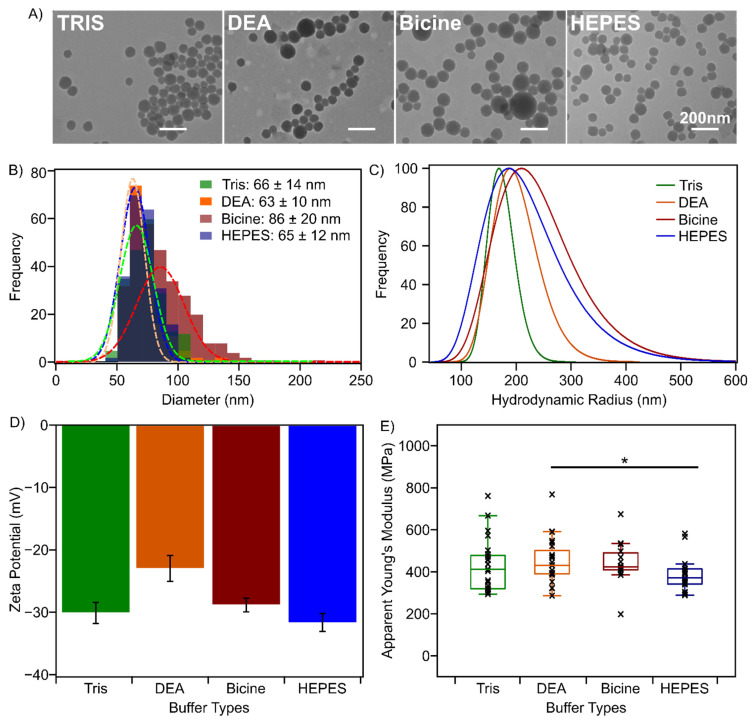
Characterization of PSeNP. (**A**) TEM images of PSeNP synthesized in Tris, DEA, Bicine, and HEPES (left to right). (**B**) Size distribution from TEM images, (**C**) hydrodynamic radius from DLS measurement, and (**D**) zeta potential (measured in 10 mM Tris, pH 7.5) of PSeNP synthesized in Tris (green), DEA (orange), Bicine (red), and HEPES (blue). The numbers in (**B**) are mean diameters. (**E**) Apparent Young’s modulus of ~150 nm PSeNP synthesized in the four buffers.

We hypothesized from previous work [15] that the presence of primary amine groups in the synthesis solvent affects the growth of polyserotonin nanoparticles (PSeNP), similar to what was observed for polydopamine nanoparticles [10]. In the current work, PSeNP were synthesized in four different buffers: Tris, DEA, Bicine, and HEPES, which were chosen to represent solvent systems containing primary (Tris), secondary (DEA), and tertiary amines (Bicine and HEPES) (Scheme 1). The spherical shapes of PSeNP were first verified using TEM (Figure 1A). The diameters were found to be 66 ± 14 nm when synthesized in Tris, 63 ± 10 nm in DEA, 86 ± 20 nm in Bicine, and 65 ± 12 nm in HEPES (Figure 1B). Consistent with what we observed in our prior work (i.e., synthesis at 60 °C for 2 h), the modified synthetic route in the current work (see Methods) generated smaller nanoparticles in the Tris buffer than those synthesized in Bicine (Figure 1A,B). However, our TEM data also show that nanoparticles synthesized in the presence of DEA and HEPES have similar sizes to those synthesized in the Tris buffer. Due to the localized nature of TEM measurements, DLS measurements were performed for a more global size distribution and also to monitor the stability in solution [19]. The DLS hydrodynamic diameters significantly increase to 167 ± 25, 189 ± 40, 211 ± 66, and 191 ± 65 nm for PSeNP synthesized in Tris, DEA, Bicine and HEPES, respectively (Figure 1C). The differences between the distributions of TEM and DLS intensity are a consequence of the differences in experimental conditions, i.e., TEM was under vacuum while DLS was in a solution and measured the hydrodynamic size rather than the real size. The synthesis in Tris resulted in the smallest polydispersity index (PDI) of 0.02, hence a more uniform size distribution, while those synthesized in DEA (PDI = 0.06), Bicine (PDI = 0.11) and HEPES (PDI = 0.20) resulted in more heterogeneous particle sizes. This could impact the size measurement obtained from DLS (as larger particles scatter much more than smaller ones) compared to TEM [20].

Our DLS data further show that, in the presence of DEA and HEPES, larger nanoparticles were formed compared to those synthesized in the presence of Tris (Figure 1C). To verify that the DLS measurement was not the result of nanoparticle aggregation, we measured the surface zeta potential of the PSeNP suspensions. Figure 1D confirms that the nanoparticles synthesized in different buffer systems form relatively stable suspensions (<−20 mV) at pH 7.5. The surface zeta potentials of PSeNP synthesized in Tris, −31 ± 2 mV have very similar values to those synthesized in both HEPES (−32 ± 1 mV) and Bicine (−29 ± 1 mV), suggesting that the observed size difference in DLS is not due to aggregation and that nanoparticles synthesized in Tris are on average smaller than those made in other buffer systems. However, our data also show that, although the presence of Tris can prevent the formation of larger particles, resulting in a more monodisperse nanoparticle size distribution, smaller nanoparticles (<100 nm) can still form independent of the buffer system used. It is also important to note that the modified synthesis method reported in this work resulted in more stable nanoparticle suspensions, ~−30 mV vs. −12 ± 5 mV (when compared to our prior work in ref. [15]), pointing to the significant contribution of the synthesis method to the surface properties of the nanoparticles, which in turn greatly affects the nanoparticle’s stability in suspension. Furthermore, our previous and current data imply that the synthetic method used for nanoparticle formation, rather than the buffer system, has a more significant impact on both the size and surface charge of the resulting PSeNP.

As the mechanical properties of a nanomaterial can affect its biological performance [21,22,23], we used atomic force microscopy (AFM) quantitative imaging to determine the elasticity (reported here as the apparent Young’s modulus) of PSeNP synthesized in different buffers. For this analysis, PSeNP of similar height profiles, ~150 nm, were particularly selected and analyzed, as it was previously shown for polydopamine nanoparticles, that elasticity is size dependent [24]. While the apparent Young’s modulus we obtained for PSeNP (~400 MPa) is comparable to the stiffness of polydopamine nanoparticles (~365 MPa) of similar size, we did not observe a significant difference in the Young’s modulus of the particles as a function of the buffer system (Figure 1E) [24]. The similarity in elasticity values, falling within a few hundred MPa range, suggests that the nature of the polymer that formed the nanoparticles and/or their assembly process is not significantly affected by the presence of buffer molecules in the solvent. Similarly, an EPR analysis showed no difference in g-values (2.0038–2.0042) across all four buffer types further indicating the formation of the same semiquinone radical species, regardless of the buffer used (Figure S1) [10,25].

### 3.2. Buffer Components Can Become Incorporated into the Nanoparticle Framework

**Figure 2:**
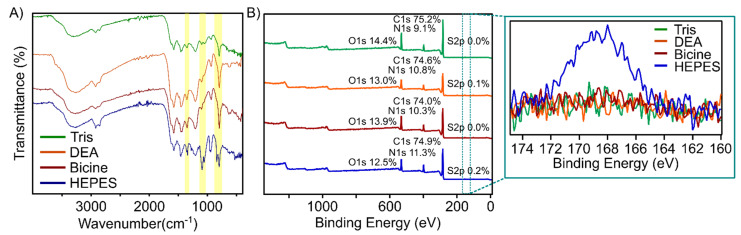
(**A**) FTIR spectra of PSeNP synthesized in Tris (green), DEA (orange), Bicine (red), and HEPES (blue) with additional adjacent peaks for HEPES in the highlighted region. (**B**) XPS survey scan of PSeNP synthesized in Tris (green), DEA (orange), Bicine (red), and HEPES (blue) with respective compositions of O 1s, N 1s, C 1s, and S2p and high-resolution scan of S2p (zoomed-in image).

The chemical composition of PSeNP was investigated using Fourier-transform infrared (FTIR) spectroscopy. The FTIR spectra of PSeNP synthesized in the different buffers display the characteristic peaks for PSeNP at >1500 cm^−1^ [15,26]. However, nanoparticles synthesized in HEPES showed additional peaks at 1369, 1056, and 838 cm^−1^ (Figure 2A). Peaks at 1369 and 1056 cm^−1^ can be assigned to S=O stretching from the sulfur group of HEPES, whereas the last peak may be caused by di-substituted C–H bending. HEPES, out of the four buffer components, has a unique sulfur atom that allows this difference to be identified, indicating that buffer molecules may be incorporated into the framework during nanoparticle formation. To further validate the presence of solvent molecules in PSeNP, an elemental surface analysis via X-ray photoelectron spectroscopy (XPS) was performed. Figure 2B confirms that the nanoparticles obtained from all buffer systems are made from oxidized serotonin, as shown by the ~3–5% increase in oxygen (vs. serotonin monomer with only 9.5% O) [15]. Furthermore, high-resolution XPS showed an increase in oxygen-containing functional groups and oxidized nitrogen functionalities (Figure S2). Though the survey spectrum of PSeNP synthesized from different buffer types showed no difference in the atomic composition of oxygen, nitrogen, and carbon, the presence of a sulfur peak (blue curve in the zoomed-in image) was observed in the high-resolution scan of S2p XPS analysis from nanoparticles made in HEPES (Figure 2B). Together with our FTIR results, this provides evidence of HEPES molecules incorporated into the nanoparticle during formation. The incorporation of solvent molecules via non-covalent interactions with oligomers of dopamine was also observed in the formation of polydopamine nanoparticles [27,28]. Here, our data suggest that this could also occur during PSeNP formation. Similarly, other buffer components could also be non-covalently incorporated during the formation of serotonin nanoparticles.

### 3.3. Density Functional Theory Calculations Support Incorporation of Buffer Molecules into the Nanoparticle Framework

**Figure 3:**
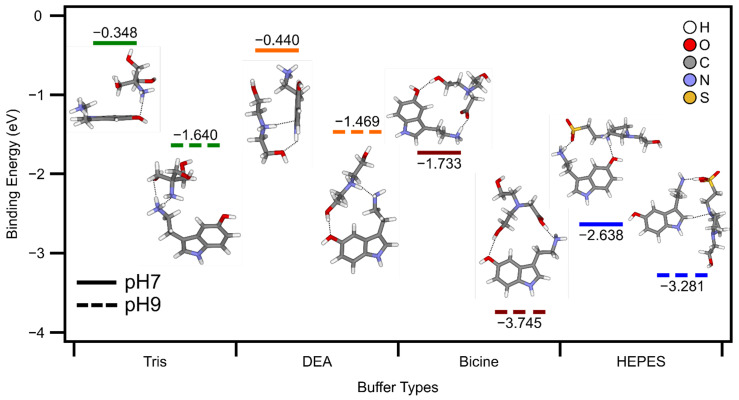
Density functional theory (DFT) simulations of the binding energy interaction between a serotonin monomer and a monomer of each buffer type (Tris, DEA, Bicine, HEPES) in pH 7 (solid line) and pH 9 (dotted line) with key interactions highlighted in black dots.

To glean the possible mechanisms by which buffer components could incorporate into the growing serotonin nanoparticles, the binding energy of serotonin monomers with buffer molecules was measured using density functional theory (DFT) calculations. Here, we used the structure of serotonin monomer since the mechanism of polyserotonin formation is currently unknown, and the structures of the early intermediates of nanoparticle formation have not been investigated. Possible intermolecular interactions of buffer molecules with serotonin could provide some information on how early intermediates of polyserotonin will interact with molecules present in the solvent during nanoparticle formation. The DFT calculations were performed at pH 9 since the synthesis of nanoparticles was initiated at this pH. In addition, DFT calculations were also performed at pH 7 to monitor the effect of protonation of the buffer components, considering the pKa of each buffer molecule (Tris pKa 8.1, DEA pKa 8.7, Bicine pKa 8.35, and HEPES pKa 7.5). On the other hand, the structure of serotonin should remain unchanged since its two pKa are greater than 9 (i.e., 9.97, 10.73) [29]. The binding interactions between serotonin and all four buffer molecules are more favorable at pH 9 than at pH 7 (Figure 3). As visualized by the optimized geometries shown in Figure 3, this enhanced interaction is a result of buffer molecules becoming deprotonated at pH 9 compared to pH 7, which then strengthens intermolecular electrostatic interactions with serotonin via charged functional groups. As nanoparticle synthesis is performed at pH 9, stronger interactions of buffer components with serotonin could signify more favorable intermolecular interactions with the early intermediates of nanoparticle formation. This scenario aligns with the observations that dopamine monomers form strong intermolecular interactions with oligomers of dopamine during the initial stages of polydopamine nanoparticle formation, resulting in dopamine being trapped within the nanoparticle framework without being involved in the polymerization process itself [27,28]. Our DFT calculations also show that all four buffer components bind favorably with serotonin monomers during the initial stages of nanoparticle synthesis at pH 9, with Bicine exhibiting the strongest binding (−3.745 eV) followed by HEPES (−3.281 eV), Tris (−1.640 eV), and DEA (−1.469 eV). A similar trend was also observed at pH 7, except that HEPES showed a more favorable binding (−2.638 eV) than Bicine (−1.733 eV). In conjunction with our FTIR and XPS measurements (presence of sulfur peak in Figure 2), this provides evidence that HEPES molecules strongly interact with serotonin, which could lead to its incorporation into the nanoparticle framework during the early stages of nanoparticle formation. We suggest that this is likely the case for other buffer components, especially Bicine. We are currently investigating whether the buffer components interact with early intermediates of polyserotonin formation, which could then lead to physical entrapment during nanoparticle growth.

### 3.4. Characterization of Polyserotonin Films

Given its structural similarities with polydopamine, polyserotonin can potentially be used as a universal film coater for different substrates [14,16,30]. We next investigated how different buffer components affected PSe film properties. Here, glass substrates were coated with PSe films under similar conditions for PSe nanoparticle formation (i.e., with purification). We showed that PSe films could successfully coat glass substrates for all the buffers tested (Figure S3). Table S1 summarizes that the films synthesized in Tris resulted in the lowest thickness (1.9 ± 0.9 nm), while those in HEPES yielded the thickest films (9.1 ± 2.1 nm). A similar trend, where Tris resulted in the thinnest film (13 ± 3 nm) and HEPES the thickest film (29 ± 6 nm), was observed when films were synthesized without purification, i.e., particles formed on day 1 were not filtered (Figure S4).

We then calculated the surface energy (with dispersive and polar components) of PSe film-coated glass surfaces using the static contact angles for three different liquids (water, glycerol, and diiodomethane) (Figure S5). Table S1 shows that for all buffer systems, the PSe film coating makes glass more hydrophilic, decreasing the water contact angle by over 15° and increasing the surface energy of glass from ~47 to ~61 mJ/m^2^. It is important to note that although the PSe film increased the overall surface energy of the glass, there is a higher contribution from the increase in the dispersive energy component than the polar energy component (Table S1). This is significantly different from polydopamine films, where an increase in surface energy was largely driven by increasing polar energy component, independent of the substrate used [31,32].

**Figure 4:**
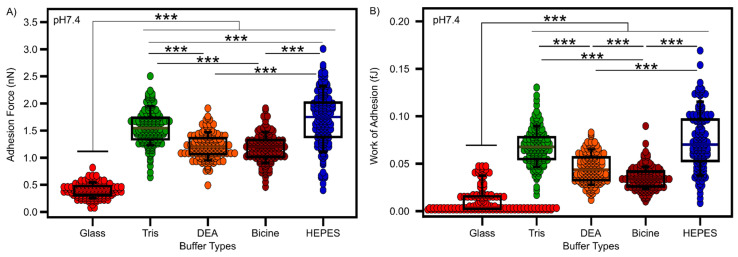
Comparison of PSe film synthesized in different buffer systems measured with APTES-functionalized AFM cantilevers. (**A**) adhesion force (n = 153) on glass (0.4 ± 0.1 nN), Tris (1.6 ± 0.3 nN), DEA (1.2 ± 0.2 nN), Bicine (1.2 ± 0.2 nN), and HEPES (1.8 ± 0.5 nN) and (**B**) work of adhesion (n = 145) on glass (0.01 ± 0.01 fJ), Tris (0.07 ± 0.02 fJ), DEA (0.04 ± 0.02 fJ), Bicine (0.03 ± 0.01 fJ), and HEPES (0.07 ± 0.03 fJ) measured at pH 7.5. One-way ANOVA was used to determine *p*-values. *** *p* ≤ 0.001.

In addition to surface energies, we also measured the interaction between a nano-sized probe of an AFM tip and the surface of PSe films under the different buffer conditions to gain insights into the adhesive properties of the films. Since polyserotonin has a negatively charged surface, we used an amine-functionalized AFM tip to probe the interactions. Figure 4 shows that both the adhesion force (Figure 4A) and work of adhesion (Figure 4B) increased when the glass was coated with the PSe film, regardless of the buffer used. However, we did observe stronger interactions with both Tris and HEPES compared with DEA and Bicine. Since the probe was functionalized with a positive charge, both the adhesion force and work of adhesion suggest that polyserotonin films synthesized in Tris and HEPES have a more negative surface compared to those synthesized in DEA or Bicine, which is consistent with the more negative zeta potential observed for nanoparticles formed in Tris and HEPES (Figure 1D).

## 4. Conclusions

Polyserotonin nanoparticles (PSeNP) were synthesized in four buffer systems (Tris, DEA, Bicine, and HEPES). We provide evidence that HEPES molecules can be incorporated into the nanoparticle network, most likely by intermolecular entrapment when oligomers/polymers collapse to form particles and/or films. Since HEPES can become incorporated, we posit that other buffer components are also likely to become trapped during nanoparticle assembly based on DFT calculations, as well as by the different surface properties of PSeNP synthesized using different buffer systems. PSeNP formed in HEPES and Tris show the most negative zeta potential, while PSe films formed in the presence of Tris and HEPES yielded the highest adhesion force and work of adhesion with a positively-charged probe. Furthermore, we showed that Tris could prevent the formation of larger nanoparticles, resulting in the most monodisperse nanoparticle suspension. Tris also prevents the formation of thicker PSe films on glass. Though solvent molecules can become incorporated during the self-assembly of polyserotonin, they do not affect the nanoparticle elasticity and the type of radical species present within the polymer network. This suggests that, in general, the nature of polymer formation, as well as the packing of polymers during the self-assembly process is not largely affected by the incorporation of buffer components.

## Supplementary Materials

The following supporting information can be downloaded at: https://www.mdpi.com/article/10.3390/nano12122027/s1, Figure S1: EPR spectrum of PSeNP synthesized in all buffers studied, Figure S2: XPS high resolution of O1s, N1s, C1s, Figure S3: AFM image and cross section of PSe film prepared under similar conditions as PSeNP, Figure S4: AFM image and cross section of PSe film prepared without purification, Figure S5: Contact angle images of PSe films, Figure S6: UV-vis spectra of PSeNP, Figure S7: TEM images of PSe extracted 45 min after heating, Figure S8: AFM topography, adhesion, and elasticity maps of PSeNP synthesized across all four buffers studied; Table S1: Characterization of PSe films formed in different buffer types on glass coverslip.
