# Supplementary material for: Buffer Components Incorporate into the Framework of Polyserotonin Nanoparticles and Films during Synthesis
Source: Nanomaterials (Basel). 2022 Jun 13;12(12):2027. doi: 10.3390/nano12122027 (PMC9227592; doi:10.3390/nano12122027)
Supplement: Supplementary file 1 [file nanomaterials-12-02027-s001.zip › nanomaterials-1726331-supplementary.pdf]

Supplementary Materials

Buffer Components Incorporate into the Framework of Polyserotonin Nanoparticles and Films during Synthesis

Keuna Jeon [1,2], Justin Andrei Asuncion [1], Alexander Lucien Corbett [1,2], Tiange Yuan [1], Meera Patel [1,2], Nesha May Octavio Andoy [1], Christian Titus Kreis [1], Oleksandr Voznyy [1,2] and Ruby May Arana Sullan [1,2,*]

[1] Department of Physical and Environmental Sciences, University of Toronto Scarborough, 1065 Military Trail, Toronto, ON, M1C 1A4, Canada; ruby.sullan@utoronto.ca
[2] Department of Chemistry, University of Toronto, 80 St. George St., Toronto, ON, M5S 3H6, Canada
* Correspondence: ruby.sullan@utoronto.ca

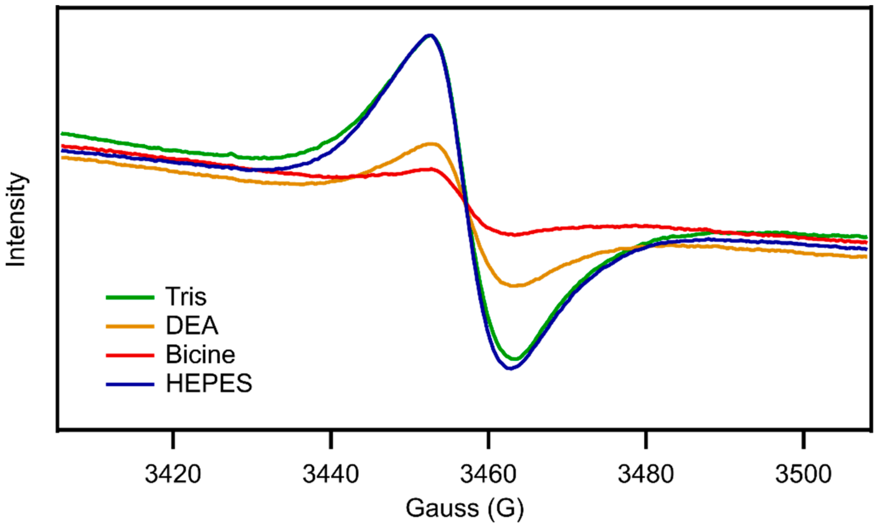

Figure S1. Electron paramagnetic resonance (EPR) spectrum of PSeNP synthesized in Tris (green, 2.0042 ± 0.0004), DEA (yellow, 2.0042 ± 0.0003), Bicine (red, 2.0038 ± 0.0004), and HEPES (blue, 2.0041 ± 0.0003).

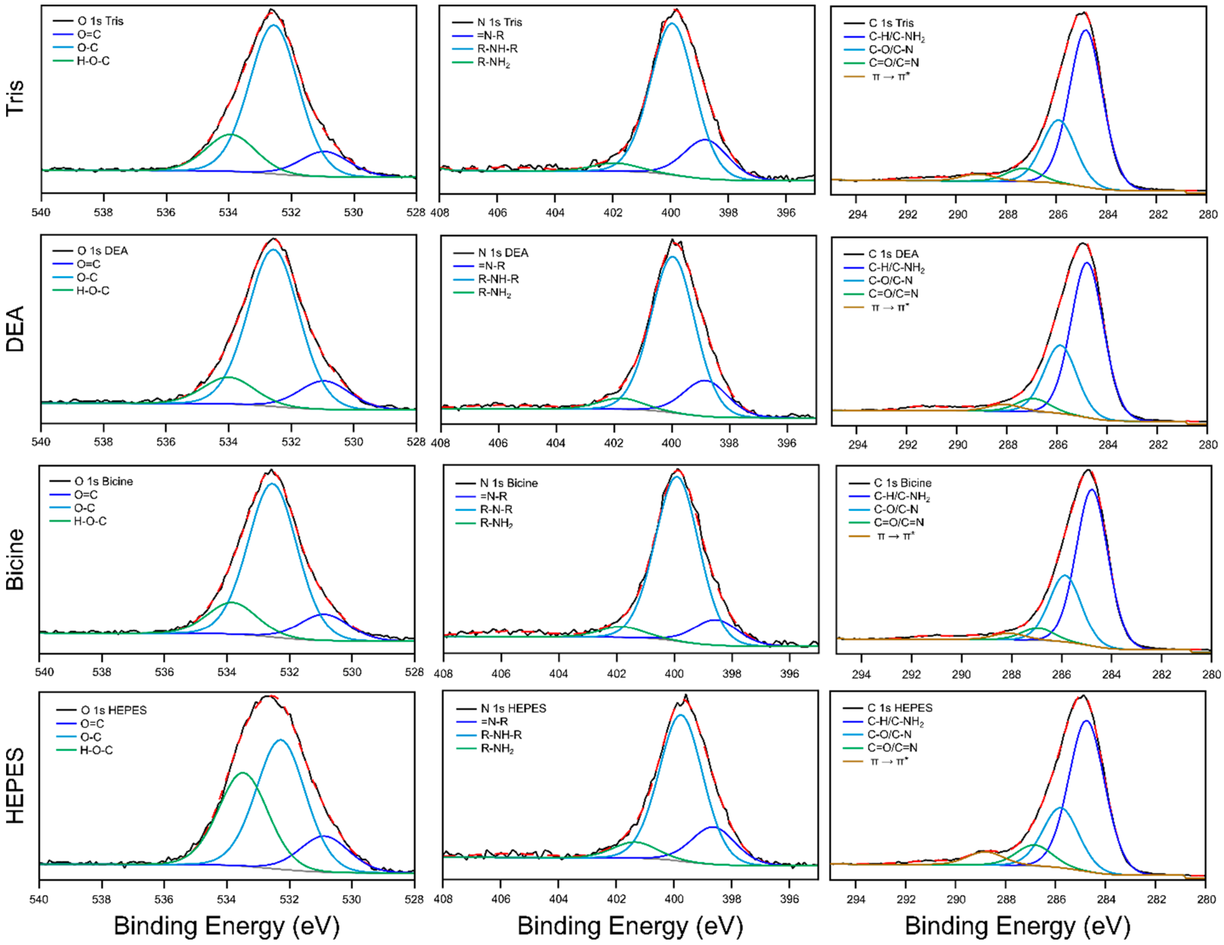

Figure S2. X-ray photoelectron spectroscopy (XPS) high-resolution spectra showing regions of O 1s (left panel), N1s (middle panel), and C 1s (right panel) of PSeNP synthesized in Tris, DEA, Bicine, and HEPES (top to bottom).

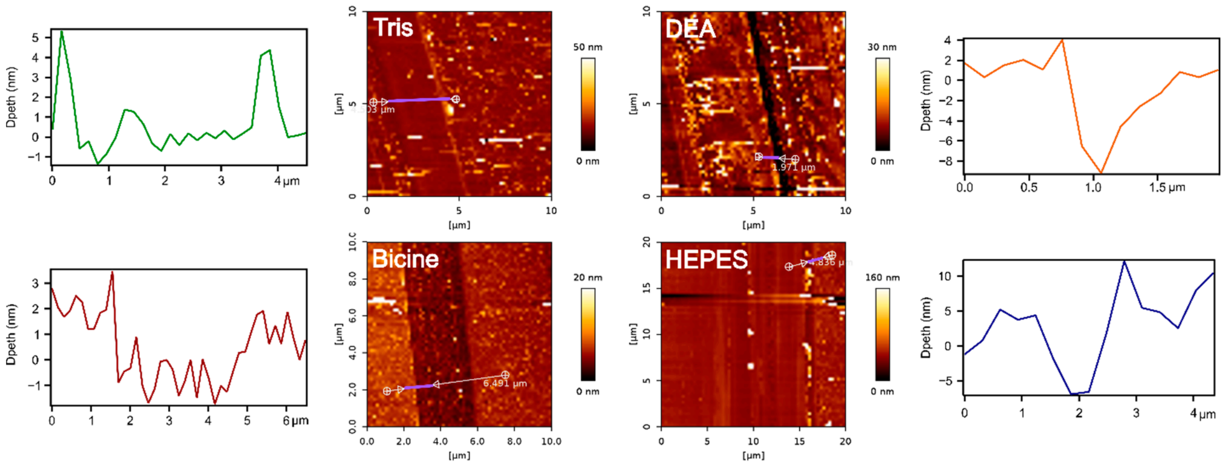

Figure S3. AFM image and corresponding cross section of PSe films synthesized from different buffer to determine the thickness of the film.

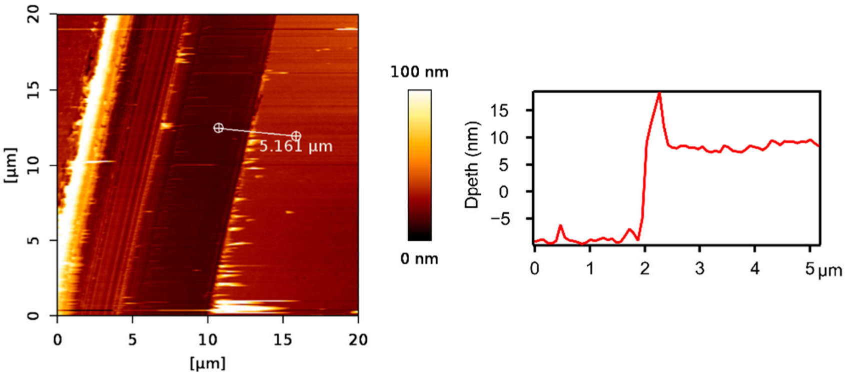

Buffer Type	Film Thickness (nm)
Tris	13 ± 3
DEA	17 ± 3
Bicine	19 ± 3
HEPES	29 ± 6

±

Figure S4. Representative atomic force microscopy (AFM) topograph of a PSe film synthesized in DEA with corresponding cross-sectional profile. Film thickness (obtained via AFM scratch test) of PSe films prepared in different buffers without purification (i.e., particles formed in Day 1 were not filtered out; also see section on PSeNP purification).

Table S1. Characterization of PSe films formed on glass coverslips in different buffers.

Buffer	Height (nm)	Contact Angle (°)			Energy (mJ/m²)		
		H_2O	DM[3]	Glycerol	Dispersive	Polar	Surface[1]
**Tris**	1.9 ± 0.9	30 ± 6	29 ± 5	27 ± 7	44.7 ± 1.7	16.2 ± 1.8	60.9 ± 2.5
**DEA**	8.1 ± 1.7	31 ± 3	28 ± 3	25 ± 4	44.9 ± 1.0	15.3 ± 1.5	60.3 ± 1.8
**Bicine**	2.5 ± 0.7	30 ± 5	31 ± 1	25 ± 2	43.6 ± 0.4	16.9 ± 0.4	60.6 ± 0.6

**HEPES**	9.1 ± 2.1	24 ± 7	30 ± 5	21 ± 2	44.2 ± 1.5	17.6 ± 1.0	61.7 ± 1.9
**control**[2]	-	45 ± 7	52 ± 6	46 ± 8	33.2 ± 2.8	13.6 ± 3.5	46.8 ± 4.5

[1] Surface energy calculated using Owens-Wendt-Rabel-Kaelble (OWRK) method.[2] no film. [3]diiodomethane

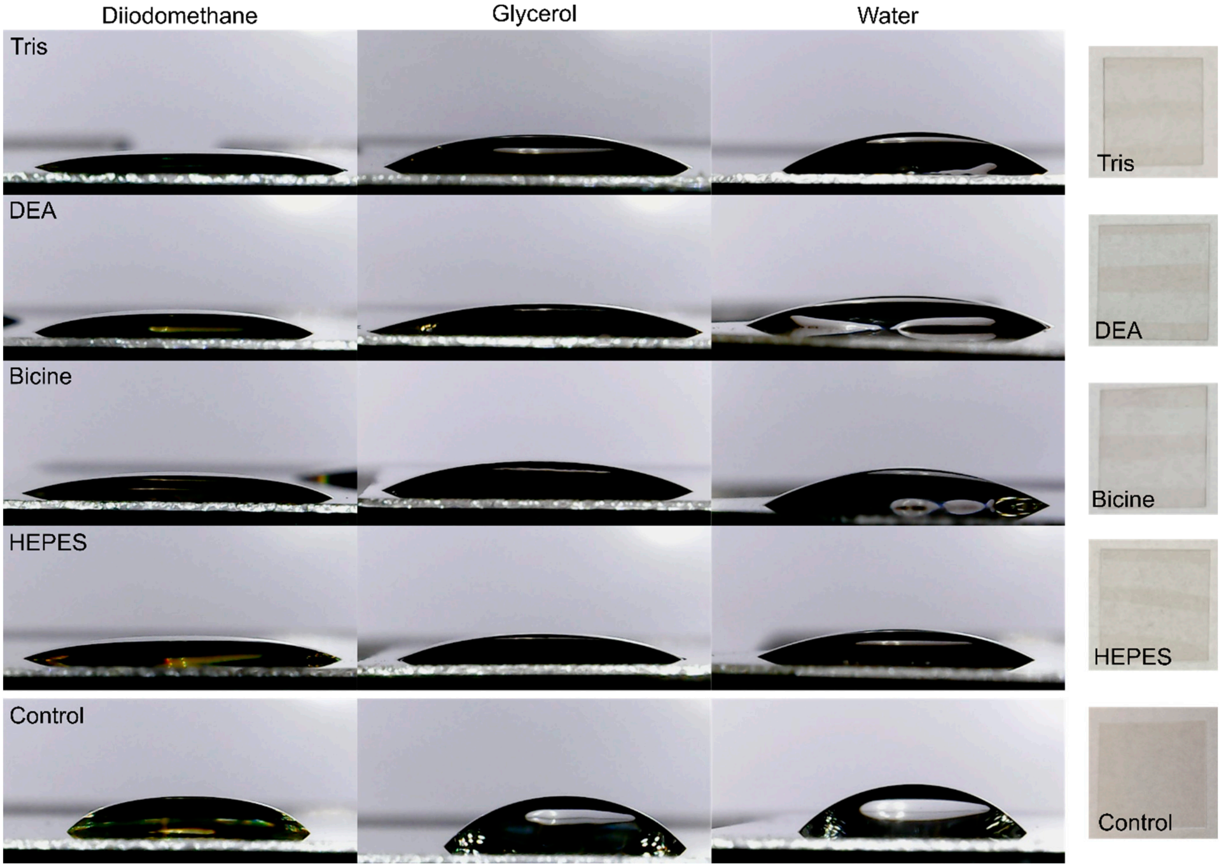

Figure S5. Contact angle images of diiodomethane, glycerol, and water on PSe films formed on glass coverslips. A panel of alternating strips of PSe films formed on the glass coverslips on the right.

Purification of Polyserotonin nanoparticles (PSeNP)

Polyserotonin nanoparticles (PSeNP) were synthesized in different buffer systems (Tris, DEA, Bicine, and HEPES) and the growth of PSeNP were monitored via UV-Vis. An increase of the signature peak at around 475 nm was observed in all four buffers after heating (Figure S6A) and after the purification process (Figure S6B). This signature UV-vis peak doesn't change across different buffer systems and coincides with PSeNP synthesized from phosphate buffer[1] and water[2]. The size of PSeNP before and after purification was also compared under TEM to validate the purification process (Figure S7). After the 45-minute heating, multiple spheres of PSe

that were merged together were extracted and the reaction was allowed to continue for an additional 24hrs (in the dark) for more uniform growth of PSeNP (Figure 1A).

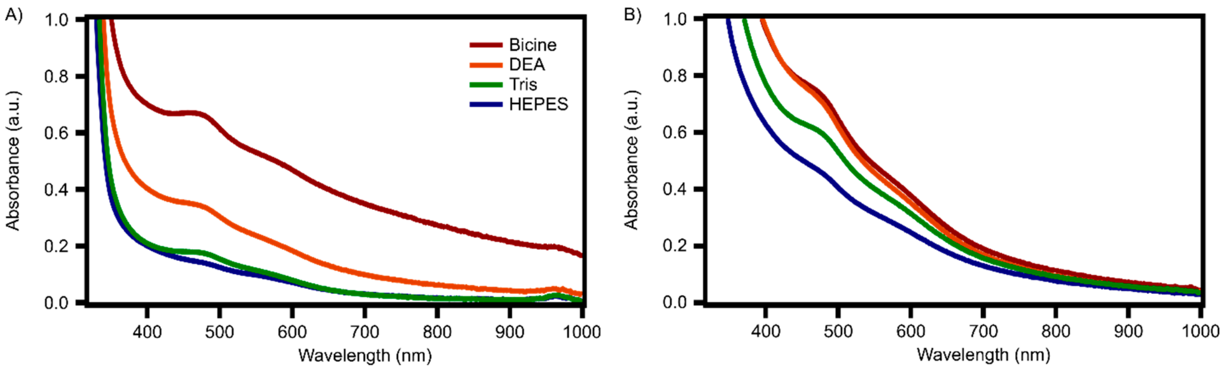

Figure S6. UV-vis spectra of PSe *after* A) 45 min of heating and B) 1 day of incubation, before particles synthesized in Tris (green), DEA (orange), Bicine (red), and HEPES (blue) buffers were centrifuged and collected.

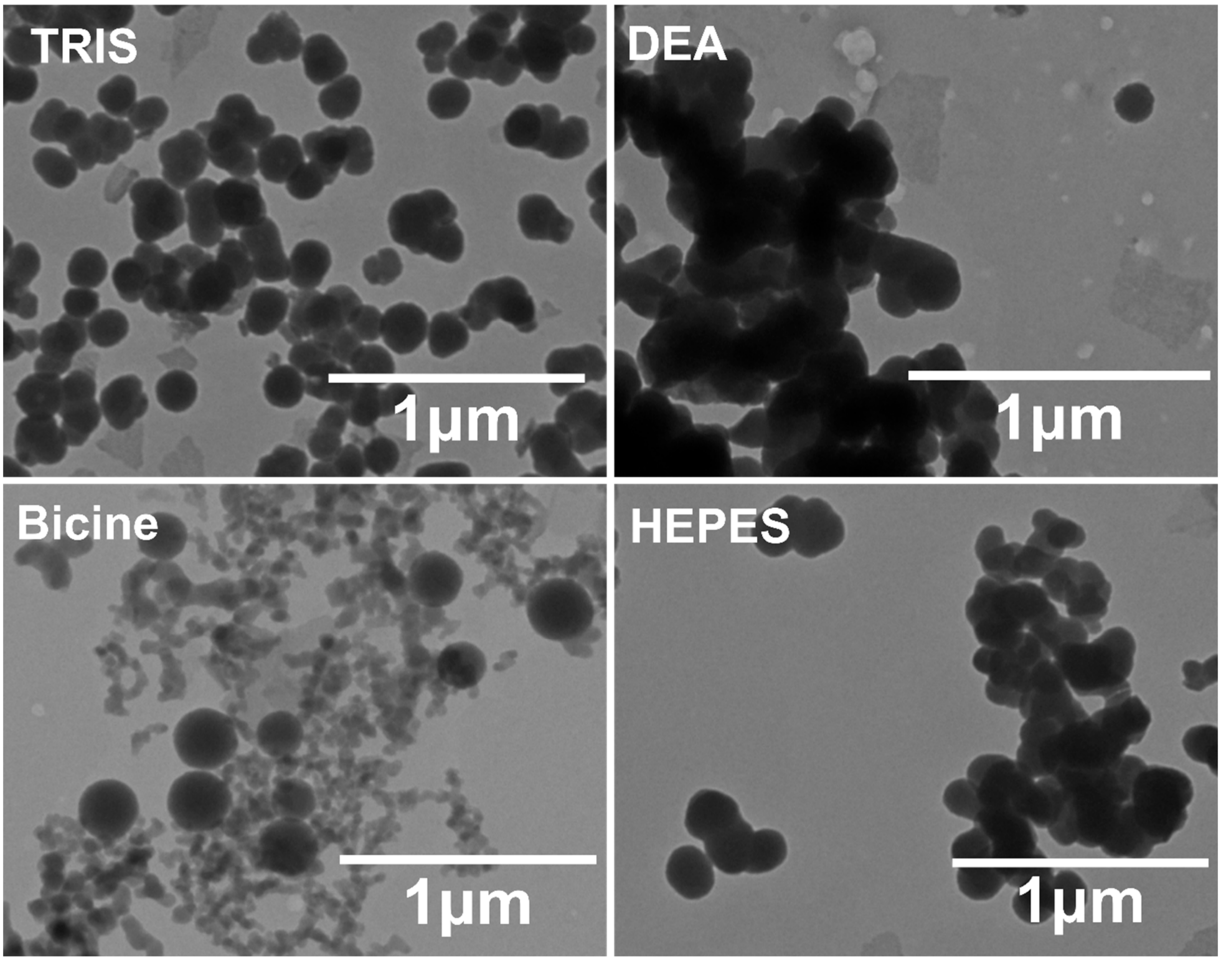

Figure S7. TEM images of PSeNP synthesized in different buffers (Tris, DEA, Bicine, HEPES) pelleted out right after 45min of heating.

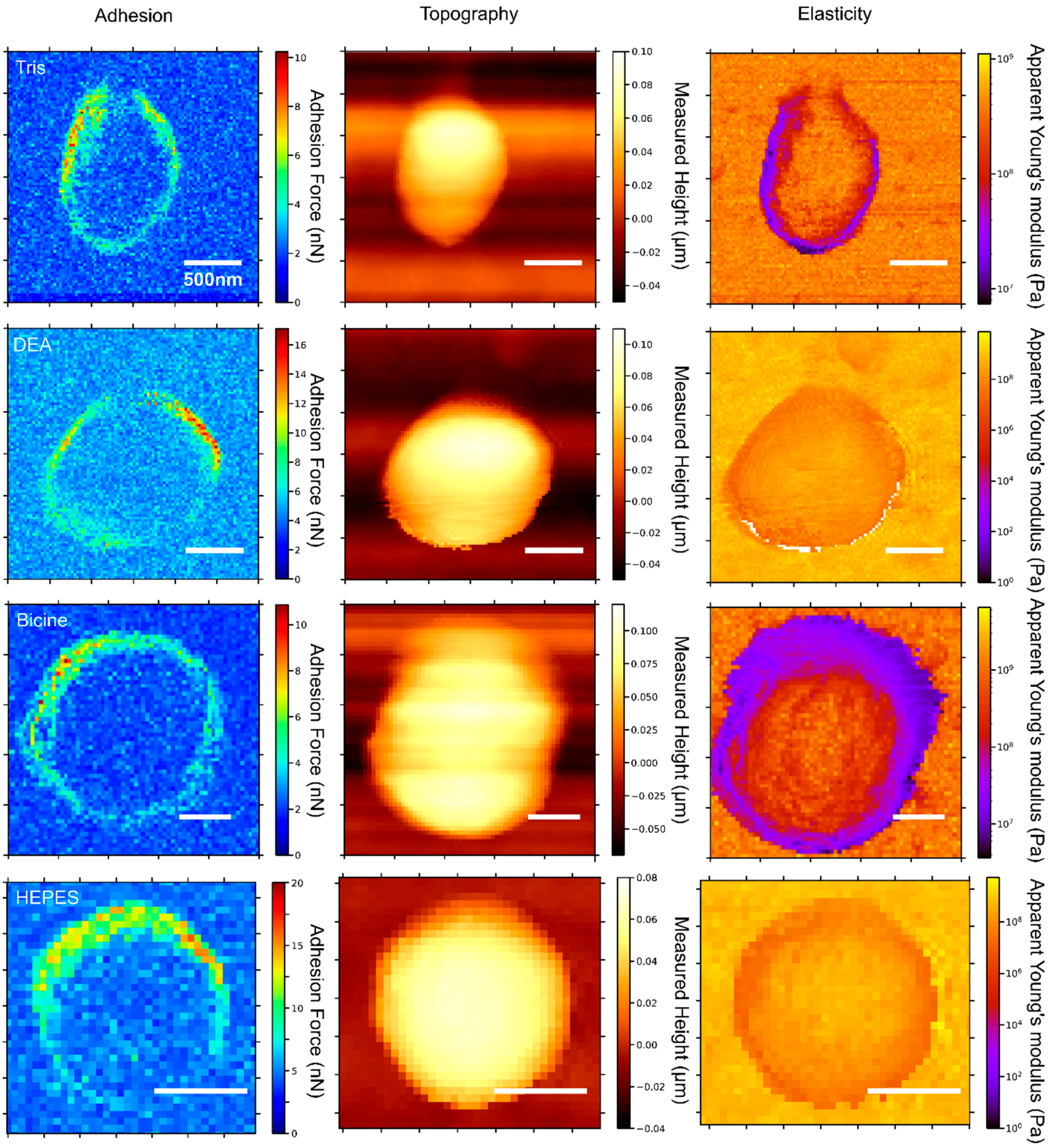

Figure S8. AFM adhesion (left), topography (middle), and elasticity (right) quantitative imaging maps of PSeNP synthesized in Tris, DEA, Bicine, and HEPES (top to bottom) Scale bar is 500nm.

References:

1. Nakatsuka, N.; Hasani-Sadrabadi, M.M.; Cheung, K.M.; Young, T.D.; Bahlakeh, G.; Moshaverinia, A.; Weiss, P.S.; Andrews, A.M. Polyserotonin Nanoparticles as Multifunctional Materials for Biomedical Applications. *Acs Nano* **2018**, *12*, 4761-4774, doi:10.1021/acsnano.8b01470.
2. Jeon, K.; Andoy, N.M.O.; Schmitt, C.W.; Xue, Y.L.; Barner, L.; Sullan, R.M.A. Size-controlled synthesis of bioinspired polyserotonin nanoparticles with free radical scavenging activity. *J Mater Chem B* **2021**, *9*, 634-637, doi:10.1039/d0tb02383c.
